# A rare case of cystic hygroma of the neck

**DOI:** 10.11604/pamj.2022.43.83.37032

**Published:** 2022-10-18

**Authors:** Surya Besant Natarajan, Krishna Prasanth Baalann

**Affiliations:** 1 Department of Community Medicine, Sree Balaji Medical College and Hospital, Bharath Institute of Higher Education and Research Institute, Chennai, India

**Keywords:** Swelling, cystic lymphangioma, lymphatic malformation

## Image in medicine

A cystic hygroma also referred to as cystic lymphangioma or macrocystic lymphatic malformation is an abnormal benign growth that typically occurs in the head or neck of the baby. This happens when there may be a defect in the formation of lymphatic vessels. Lymph is the content of those cyst-like cavities in the malformation. The lymphatic malformations are either macrocystic (large cysts) or microcystic (small cysts). The disorder usually develops while the foetus remains in the uterus but may also appear after birth. It commonly presents as a mass within the neck found at birth or discovered later in an infant following an upper respiratory tract infection. Cystic hygromas are often related to a nuchal lymphangioma or a fetal hydrops. Also, they are frequently associated with down syndrome, Turner syndrome, and Noonan syndrome. The incidence of nuchal cystic hygroma is about 1/6000 at birth and about 1/750 in miscarriage. Antenatally, amniocentesis is required to diagnose if cystic hygroma is suspected during USG. After birth, USG, X-rays, and computed tomography (CT) scans are used for diagnostic purposes. The prognosis is commonly considered poor. Treatment options for removal of cystic hygroma are by surgery or by using sclerosing agents. A newborn presented with swelling in the neck region at birth. On evaluation, it was found to be a case of congenital cystic hygroma of the neck. The child was an ideal candidate for surgery, and therefore the parents were explained about the various treatment modalities and advised to follow up.

**Figure 1:**
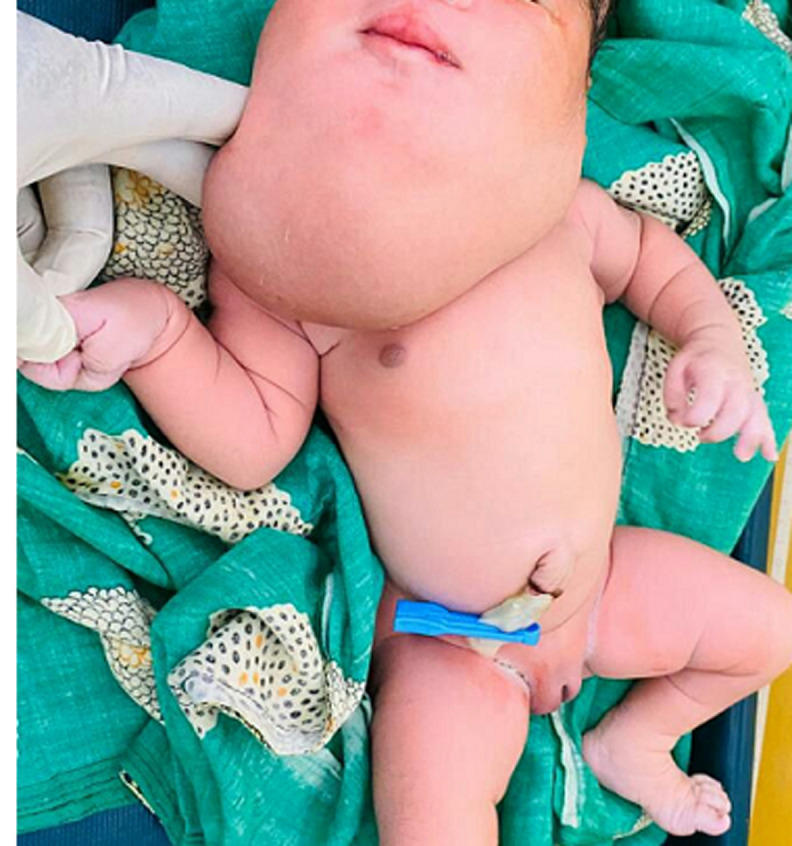
swelling in the neck region

